# One-step stromal vascular fraction therapy in osteoarthritis with tropoelastin-enhanced autologous stromal vascular fraction gel

**DOI:** 10.3389/fbioe.2024.1359212

**Published:** 2024-02-12

**Authors:** Junjun Yang, Xin Wang, XueBao Zeng, Rong Wang, Yanming Ma, Zhenlan Fu, Zu Wan, Zhi Wang, Liu Yang, Guangxing Chen, Xiaoyuan Gong

**Affiliations:** ^1^ Center for Joint Surgery, Southwest Hospital, Army Medical University (Third Military Medical University), Chongqing, China; ^2^ Key Laboratory of Biorheological Science and Technology, Ministry of Education College of Bioengineering, Chongqing University, Chongqing, China; ^3^ Chongqing Yan Yu Medical Beauty Clinic, Chongqing, China

**Keywords:** stromal vascular fraction gel, osteoarthritis, tropoelastin, mesenchymal stem cells, anoikis

## Abstract

**Background:** Osteoarthritis (OA) is a debilitating degenerative joint disease, leading to significant pain and disability. Despite advancements, current regenerative therapies, such as mesenchymal stem cells (MSCs), face challenges in clinical efficacy and ethical considerations. This study aimed to evaluate the therapeutic potential of stromal vascular fraction gel (SVF-gel) in comparison to available treatments like hyaluronic acid (HA) and adipose-derived stem cells (ADSCs) and to assess the enhancement of this potential by incorporating tropoelastin (TE).

**Methods:** We conducted a comparative laboratory study, establishing an indirect co-culture system using a Transwell assay to test the effects of HA, ADSCs, SVF-gel, and TE-SVF-gel on osteoarthritic articular chondrocytes (OACs). Chondrogenic and hypertrophic markers were assessed after a 72-hour co-culture. SVF-gel was harvested from rat subcutaneous abdominal adipose tissue, with its mechanical properties characterized. Cell viability was specifically analyzed for SVF-gel and TE-SVF-gel. The *in vivo* therapeutic effectiveness was further investigated in a rat model of OA, examining MSCs tracking, effects on cartilage matrix synthesis, osteophyte formation, and muscle weight changes.

**Results:** Cell viability assays revealed that TE-SVF-gel maintained higher cell survival rates than SVF-gel. In comparison to the control, HA, and ADSCs groups, SVF-gel and TE-SVF-gel significantly upregulated the expression of chondrogenic markers COL 2, SOX-9, and ACAN and downregulated the hypertrophic marker COL 10 in OACs. The TE-SVF-gel showed further improved expression of chondrogenic markers and a greater decrease in COL 10 expression compared to SVF-gel alone. Notably, the TE-SVF-gel treated group in the *in vivo* OA model exhibited the most MSCs on the synovial surface, superior cartilage matrix synthesis, increased COL 2 expression, and better muscle weight recovery, despite the presence of fewer stem cells than other treatments.

**Discussion:** The findings suggest that SVF-gel, particularly when combined with TE, provides a more effective regenerative treatment for OA by enhancing the therapeutic potential of MSCs. This combination could represent an innovative strategy that overcomes limitations of current therapies, offering a new avenue for patient treatment. Further research is warranted to explore the long-term benefits and potential clinical applications of this combined approach.

## 1 Introduction

Osteoarthritis (OA) is the most common degenerative joint disorder and it is the leading cause of joint pain and stiffness, which significantly affects the quality of life of millions of people worldwide (Foster et al., 2023). The primary pathological features of OA include the progressive loss of articular cartilage, osteophyte formation, subchondral bone remodeling, and synovial inflammation (Yao et al., 2023). These pathological alterations result in chronic pain, joint stiffness, and impaired mobility, significantly impacting the patient’s quality of life. Current conservative strategies for OA interventions are primarily symptomatic, focusing on pain management and improving joint function (Siemi et al., 2017; Cho et al., 2021), However, these strategies often fail to fully restore joint function and do not prevent disease progression (Englund, 2023). Mesenchymal stem cells (MSCs), known for the ability to renew themselves and transform into different cell types, have become a promising tool in regenerative medicine, including OA treatment (Galipeau and Sensébé, 2018). Specifically, MSCs therapy has demonstrated potential in OA treatment, showing improvements in pain, function, and even signs of cartilage repair in pre-clinical and clinical studies (Yu et al., 2022). However, MSCs therapy also faces significant challenges. Acquiring enough MSCs for treatment requires extensive *in vitro* expansion, which is expensive, time-consuming, and requires specialized laboratory equipment and growth factors (Johnson et al., 2012). Long culture periods can also lead to cells aging and losing their MSCs characteristics. Survival and retention of the transplanted MSCs within the harsh joint environment is another challenge. The OA joint, often inflamed, may provoke anoikis of transplanted MSCs, and reducing their therapeutic effects (Wang and Cao, 2014; Hwang et al., 2021).

Stromal vascular fraction gel (SVF-gel) is a bio-product created from adipose tissue, obtained typically through liposuction (Park et al., 2022). The tissue undergoes enzymatic digestion, centrifugation, and filtration to yield a gel-like substance rich in cellular components such as pre-adipocytes, endothelial cells, and pericytes. The key constituents are MSCs, renowned for their regenerative capabilities (Cai Y. et al., 2023). Additionally, SVF-gel retains extracellular matrix (ECM) proteins, providing a natural scaffold that fosters cell adhesion, survival, and differentiation (Ye et al., 2021). In the field of plastic surgery, SVF-gel has been effectively utilized to enhance tissue volume, promote hair growth, improve skin texture, and promote wound healing (Wang et al., 2019; Xiao et al., 2020; Cai J. et al., 2023). Building on its successful applications in plastic surgery, SVF-gel also presents potential for treating OA. Preliminary clinical studies suggested that SVF-gel showed superior outcome to microfracture, evidenced by enhanced expression of type II collagen and promoted cartilage regeneration in defect area (Li et al., 2020). In addition, the notable advantage of simple preparation for SVF-gel reduced the time between tissue collection and treatment. This also avoids potential phenotype changes and cell senescence associated with prolonged cell culture (Jiang et al., 2022). However, the cell viability of MSCs within the SVF-gel might potentially be compromised during the extraction and preparation process. Therefore, it is essential to enhance the activity and function of MSCs based on the SVF-gel foundation to facilitate better therapeutic outcomes.

Tropoelastin (TE), the monomeric precursor to elastin, plays crucial roles in providing elasticity and resilience to various tissues. Hence, it holds significant potential in tissue engineering and regenerative medicine (Mithieux et al., 2013). In this field, TE has demonstrated promise in multiple applications. For instance, due to its inherent elasticity and biocompatibility, TE is extensively used in the fabrication of biomaterial scaffolds to promote MSCs tenogenic commitment and deposition of elastin-rich matrix (Almeida et al., 2019). Moreover, a composite hydrogel synthesized from gelatin-methacryloyl and methacryloyl-substituted TE, demonstrated superior tissue adhesion, promoted nerve regeneration, and could potentially reduce the need for suturing in nerve reconstruction (Soucy et al., 2018). Our recent study demonstrated that TE could promote the performance of infrapatellar fat pad MSCs (IPFP-MSCs) and protects knee cartilage from damage in OA through enhancement of cell adhesion and activation of integrin β1/ERK/Vinculin pathway (Yang et al., 2022). In addition, we further showed that TE-pretreated adipose-derived stem cells (TE-Exo^ADSCs^) maintained the chondrocyte phenotype *in vitro* and promoted cartilage repair *in vivo* (Meng et al., 2023), potentially through altering the expression of miR-451-5p in Exo^ADSCs^. Thus, TE offers new possibilities in tissue engineering and regenerative medicine with its unique physicochemical properties and biological functions.

In light of these considerations, the present study aims to explore the therapeutic potential of TE-supplemented SVF-gel (TE-SVF-gel) in the treatment of OA in a rat model. We have extracted SVF-gel and incorporated TE, aiming to evaluate the impact of this novel treatment on the maintenance of the chondrocyte phenotype and its efficacy in mitigating OA symptoms. A group treated solely with ADSCs serves as a positive control, allowing us to assess the differential therapeutic effects. Through these investigations, we hope to shed light on the potential of TE-SVF-gel as a promising therapeutic option in the management of OA.

## 2 Materials and methods

### 2.1 Preparation of SVF-gel and TE-SVF-gel from rat abdominal subcutaneous adipose tissue

All animal experiments were conducted in accordance with the animal research committee regulations of the Army Medical University (Third Military Medical University), under approval number AMUWEC20223814. Abdominal subcutaneous fat was carefully collected from rats. The extracted fat tissue was thoroughly cleaned to remove attached synovial, blood vessel, and fibrous tissues. The fat tissue was placed into a 50 mL syringe and broken-down using PAL-Electric Fat Mincer (SUN-ZT Medical Technology Co., Ltd.), then directly transferred from the syringe into a centrifuge tube. Immediately after, the tube was spun at 2000 g for 5 min in a horizontal centrifuge. This centrifugation step was repeated three times to ensure the effective separation of the SVF-gel from the rest of the adipose tissue. The SVF-gel was collected carefully from the bottom of the centrifuge tube. The semi-fluid gel-like substance, indicating the successful preparation of SVF-gel. In the preparation of TE-SVF-gel, the rat adipose tissue was supplemented with 20 μg/mL of TE (Advanced BioMatrix) throughout the mincing and subsequent centrifugation processes, while all other aspects of the protocol remained unchanged. TE was dissolved in 0.25% glacial acetic acid (Beyotime) and diluted with normal saline.

### 2.2 Cell viability assay using calcein-AM/PI staining

The SVF-gel and TE-SVF-gel was first liquefied by adjusting the temperature to 37°C in incubator. Following liquefaction, the SVF-gel and TE-SVF-gel was added to saline solution. The mixture was then centrifuged at 300 *g* for 5 min to collect the cells. The viability of the cells was assessed using a Calcein-AM/PI staining kit (Beyotime). In brief, cells were washed with PBS and then incubated with a staining solution containing Calcein-AM and propidium iodide (PI) in phosphate-buffered saline (PBS) for 30 min at 37°C in a humidified atmosphere of 5% CO_2_. Following incubation, cells were washed twice with PBS to remove excess dye. Live cells, characterized by their ability to convert non-fluorescent calcein-AM into green-fluorescent calcein, and dead cells, identified by their permeability to PI which intercalated into DNA and RNA to emit red fluorescence, were visualized under a confocal fluorescence microscope. The number of live and dead cells was counted in three random fields per well, and the percentage of viable cells was calculated as (number of live cells/total number of cells) × 100% (Liu et al., 2020).

### 2.3 Rheological measurements of SVF-gel

To explore the printing parameters of SVF-gel, the rheologic test of SVF-gel was performed with rheometer (discovery hr-20, TA, USA), including temperature, frequency and strain sweep. A 40 mm diameter and 1.98944° cone plate geometry was used. For SVF-gel, the gap size was 100 µm in all cases. Temperature control was maintained using the bottom plate attachments with cooling supplied by a circulating chiller. Results were recorded and analyzed using TRIOS software. The storage modulus (G′) and loss modulus (G″) at 25°C were detected. The frequency sweeps were carried out in the range of 0.1–10 Hz at a constant strain of 1%. Under the strain sweeps, the strain was set between 0.01% and 500% and the scanning frequency was 1 Hz.

### 2.4 Isolation and culturing of chondrocytes from OA rat knee joints

The knee joints were carefully dissected under sterile conditions. The joint capsule was opened and the cartilage from the femoral condyles and tibial plateaus was carefully removed using a scalpel and forceps while avoiding any subchondral bone. The isolated cartilage pieces were washed three times with PBS to remove blood and debris. The clean cartilage pieces were then minced into small fragments approximately 1 mm in size. The cartilage fragments were digested in a solution of 0.2% collagenase II (Biosharp) in Dulbecco’s Modified Eagle Medium (DMEM) (Gibco) at 37°C for 6 h gentle agitation. The resulting cell suspension was filtered through a 40 μm cell strainer to remove undigested tissue pieces. The cell suspension was centrifuged at 300 *g* for 5 min to pellet the cells. The supernatant was discarded and cell pellet was resuspended in DMEM supplemented with 10% fetal bovine serum (FBS) (Gibco), 1% penicillin-streptomycin (PS) (Beyotime). The cells were seeded in a tissue culture flask and placed in a humidified incubator with 5% CO_2_ at 37°C. The culture medium was changed every 2 d. Once the cells reached 80% confluence, they were detached using 0.25% trypsin-EDTA solution and either passaged or used for subsequent experiments (Yang et al., 2020).

### 2.5 Isolation, culture, and expansion of ADSCs from rat subcutaneous adipose tissue

ADSCs were isolated from the subcutaneous adipose tissue of rats. Specifically, the adipose tissue was harvested from the abdominal region of the rats. Following harvest, the adipose tissue was minced into small pieces and then subjected to enzymatic digestion with 0.2% collagenase II (Biosharp) for the isolation of ADSCs. The resulting cell suspension was filtered to remove debris and then centrifuged to separate the ADSCs. The pellet with the ADSCs was re-suspended in DMEM/F12 (Gibco) supplemented with 10% FBS and 1% PS before seeding into culture flasks. The ADSCs were cultured in a thermostatic incubator, and their growth and morphology were regularly monitored. The culture medium was replaced every 2 days to ensure nutrient availability. Upon reaching approximately 80% confluence, the cells were detached using a trypsin-EDTA solution (Gibco) and re-seeded into new flasks for further expansion. The third passage of ADSCs were used for the subsequent experiments.

### 2.6 Establishment of indirect co-culture system for chondrocytes

To investigate the influence of various treatment approaches on osteoarthritic articular chondrocytes (OACs), an indirect co-culture system was established utilizing a Transwell setup. In this system, HA, ADSCs, SVF-gel, or TE-SVF-gel were placed in the upper compartment of Transwell, while the lower compartment was seeded with OACs. The co-culture was maintained for 72 h, after which the expression levels of chondrogenic markers (SOX-9, ACAN, and COL 2) and the hypertrophic marker (COL 10) in OACs were examined.

### 2.7 Induction of OA in rats using destabilization of the medial meniscus (DMM)

The study utilized thirty-six female Sprague Dawley (SD) rats (280 g), which were randomly divided into six groups of six rats each: Control, DMM, HA, SVF-gel, TE-SVF-gel, and ADSCs. After inducing anesthesia in the rats with sodium pentobarbital, a medial parapatellar incision was made at the knee joint. The medial meniscus was then destabilized to induce OA (Yan et al., 2023). The success of the DMM procedure was confirmed by visual inspection and palpation, ensuring instability of the medial meniscus. Upon confirmation, the incision was sutured. Postoperatively, the rats were allowed to move freely in their cages. Eight weeks following the DMM surgery, the induction of OA was considered successful. The groups receiving HA, SVF-gel, TE-SVF-gel, and ADSCs were given a single injection. Notably, the number of cells in ADSCs injection was consistent with the cell number in the SVF-gel (1 × 10^8^ cells). The progression of OA was periodically monitored, and 8 weeks post-injection, the therapeutic effects on OA were evaluated. A histological examination of the knee joints was performed at the study endpoint to confirm the induction of the disease and to assess the therapeutic effects of the various treatments.

### 2.8 Immunofluorescence (IF) staining

Rat chondrocytes were fixed with 4% paraformaldehyde for 15 min, followed by three washes with PBS. To permeabilize the cells, 0.1% Triton X-100 in PBS was added for 10 min, followed by another three PBS washes. Non-specific binding sites were blocked with 5% bovine serum albumin (BSA) (Beyotime) in PBS for 1 h. After blocking, cells were incubated with the primary antibodies against COL 2, COL 10, SOX-9, and ACAN (Abcam), all directly diluted at a ratio of 1:200, overnight at 4°C. Following this, Cells were washed thrice with PBS and then incubated for 1 h at room temperature with the secondary antibodies Ms-647 and Rb-488 (Abcam), both directly diluted at a ratio of 1:500. After another three washes with PBS, cells were stained with 4′,6-diamidino-2-phenylindole (DAPI) (Beyotime) for 5 min to visualize nuclei. Cells were then washed with PBS, mounted, and examined under a confocal microscope. Images were acquired and analyzed using the ZEN 2012.

### 2.9 Western blot analysis

Rat chondrocytes were lysed in RIPA buffer (Beyotime) supplemented with protease and phosphatase inhibitor cocktail (Beyotime). The protein concentration was determined using the BCA Protein Assay Kit (Beyotime). Equal amounts of protein (50 µg) were separated by 10% SDS-PAGE (Beyotime) and transferred to PVDF membranes (Millipore). Following transfer, the membranes were blocked with 5% BSA in TBS containing 0.1% Tween-20 (TBST) for 1 h at room temperature. The membranes were then incubated overnight at 4°C with primary antibodies (COL 2, COL 10, SOX-9, and ACAN), directly diluted at a ratio of 1:1000 in 5% BSA-TBST. The following day, after washing thrice with TBST, the membranes were incubated with horseradish peroxidase-linked secondary antibodies, directly diluted at a ratio of 1:5000 (Abcam), for 1 h at room temperature. After washing three times with TBST, the membranes were visualized using the SuperSignal West Femto Kit (Thermo Fisher). Finally, the intensity of the blots was quantified using Image Lab 3.0 software (Bio-Rad). After washing three times with TBST, the membranes were visualized using the SuperSignal West Femto Kit (Thermo Fisher). Finally, the intensity of the blots was quantified using Image Lab 3.0 software (Bio-Rad) (Huang et al., 2017).

### 2.10 Tracking of injected ADSCs in the rat knee joint cavity

After intra-articular injections of SVF-gel, TE-SVF-gel, and ADSCs, the ADSCs within the knee joint cavity were identified and tracked using the Human MSCs Analysis Kit (BD Biosciences). Synovium were carefully dissected and isolated from the rats 72 h after intra-articular injections of SVF-gel, TE-SVF-gel, and ADSCs, taking care to avoid contact with surface of the synovium. The synovium was then fixed, permeabilized, and blocked to reduce non-specific binding. Following this, the synovium was incubated with a primary antibody against the CD 90. After washing three times to remove unbound primary antibodies, tissues were incubated with a fluorescent secondary antibody at room temperature for 1 h. Following another set of three washes to remove unbound secondary antibodies, the tissues were visualized under a confocal microscope. Through the aforementioned experimental procedures, the exogenous ADSCs on the surface of the synovium were tracked.

### 2.11 Micro CT imaging

The knee joints were scanned with Micro-CT (viva CT-40, ScancoMedical AG, Switzerland). Image acquisition was performed with the condition of 45 kV and 177 μA in highresolution scans (10.5 μm voxel resolution). Sagittal and coronal plane images from rat knee joint CT scans were utilized to evaluate the presence of osteophytes and signs of osteoporosis in OA. Furthermore, an analysis of bone volume to total volume (BV/TV) and trabecular thickness was conducted to further assess the bone health (Jiang et al., 2023).

### 2.12 Measurement of gastrocnemius muscle weight

Rats were euthanized, and the gastrocnemius muscle was carefully dissected from the lower hind limb on both the OA-affected and unaffected sides. All visible connective tissue was removed to ensure only muscle tissue was weighed. The muscles were then blotted to remove excess blood and moisture, weighed immediately using a precision balance, and the weight recorded. Care was taken to perform the dissection and weighing in a consistent manner across all specimens to ensure comparability of the data (Shang et al., 2024).

### 2.13 Histological analysis

Following the endpoint of the study, rat knee joints from each group were harvested and fixed in 4% paraformaldehyde, then decalcified in EDTA solution and embedded in paraffin. Sections of 5 μm were cut and mounted on glass slides. For histological analysis (Wu et al., 2019), sections were deparaffinized, rehydrated, and stained with Hematoxylin and Eosin (HE) to assess the general architecture and cellular details of the tissues. For assessment of cartilage glycosaminoglycans, a subset of sections were stained with Safranin O and Fast Green. After deparaffinization and rehydration, the sections were stained with Fast Green for 5 min, rinsed in 1% acetic acid, and then stained with Safranin O for 5 min. For immunohistochemical (IHC) analysis of COL 2, sections underwent antigen retrieval before being blocked and incubated with primary antibodies against COL 2 overnight at 4°C. This was followed by washing and incubation with a biotinylated secondary antibody, which was then detected using a standard ABC (avidin-biotin complex) system and visualized with a DAB (3,3′-diaminobenzidine) substrate, resulting in a brown precipitate at the antigen site. The sections were then counterstained with Hematoxylin, dehydrated, and mounted. All slides were independently evaluated by blinded observers, with the severity of OA and the distribution of COL 2 recorded and analyzed.

### 2.14 Statistical analysis

Data are presented as mean values ±standard deviation (SD) and subjected to analysis using GraphPad Prism 9.0 software. All *in vitro* experiments were replicated three times. Standard statistical tests including the independent-sample *t*-test and one-way ANOVA with Tukey’s *post hoc* test were employed for comparison between two distinct groups and multiple group analysis respectively, under the assumptions of normal distribution and homogeneity of variances. The concept of normal distribution refers to a frequently encountered form of continuous probability distribution characterized by a bell-shaped, symmetrical curve. Homogeneity of variances implies equal variance across all groups. For ranked data or data that did not fulfill the prerequisites of normal distribution and equal variance, non-parametric methods such as the Kruskal-Wallis test and Nemenyi test were utilized. The Kruskal-Wallis test, a non-parametric technique, is used for comparing three or more independent samples without the need for normal distribution or equal variance assumptions. Following a significant Kruskal-Wallis outcome, the Nemenyi test was utilized for pairwise comparisons. In all instances, a *p*-value of less than 0.05 was considered to demonstrate statistical significance.

## 3 Results

### 3.1 Addition of TE effectively reduced cell death during SVF-gel preparation

**FIGURE 1:**
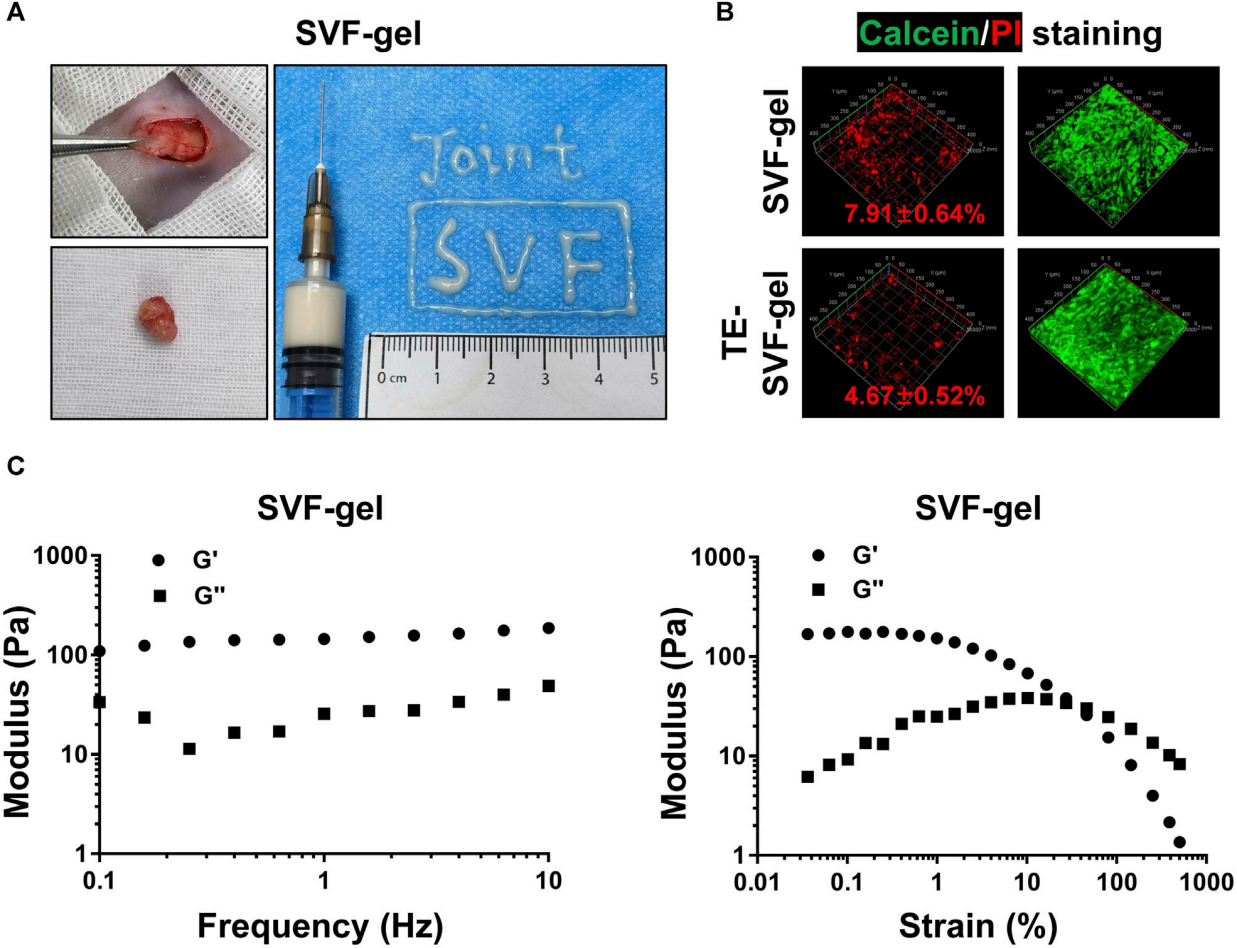
The preparation process, rheological properties, and the ability of TE to reduce the proportion of dead cells in SVF-gel. **(A)** Preparation process of SVF-gel and its injectability. **(B)** Calcein/PI staining of stromal vascular fractions (SVFs). **(C)** Frequency and strain sweep of SVF-gel.

As shown in Figure 1A, SVF-gel derived from the subcutaneous abdominal adipose tissue of rats demonstrated good fluidity and injectability. In the viability assay of the SVF-gel, it was found to contain a certain proportion of dead cells (7.91% ± 0.64%). In contrast, addition of TE in the SVF-gel during the preparation process reduced the proportion of dead cells (4.67% ± 0.52%) (Figure 1B). Furthermore, preliminary rheological experiments confirmed that the SVF-gel possesses gel-like properties (Figure 1C).

### 3.2 TE-SVF-gel effectively enhanced chondrogenic matrix expression and reduced hypertrophic differentiation in OACs

**FIGURE 2:**
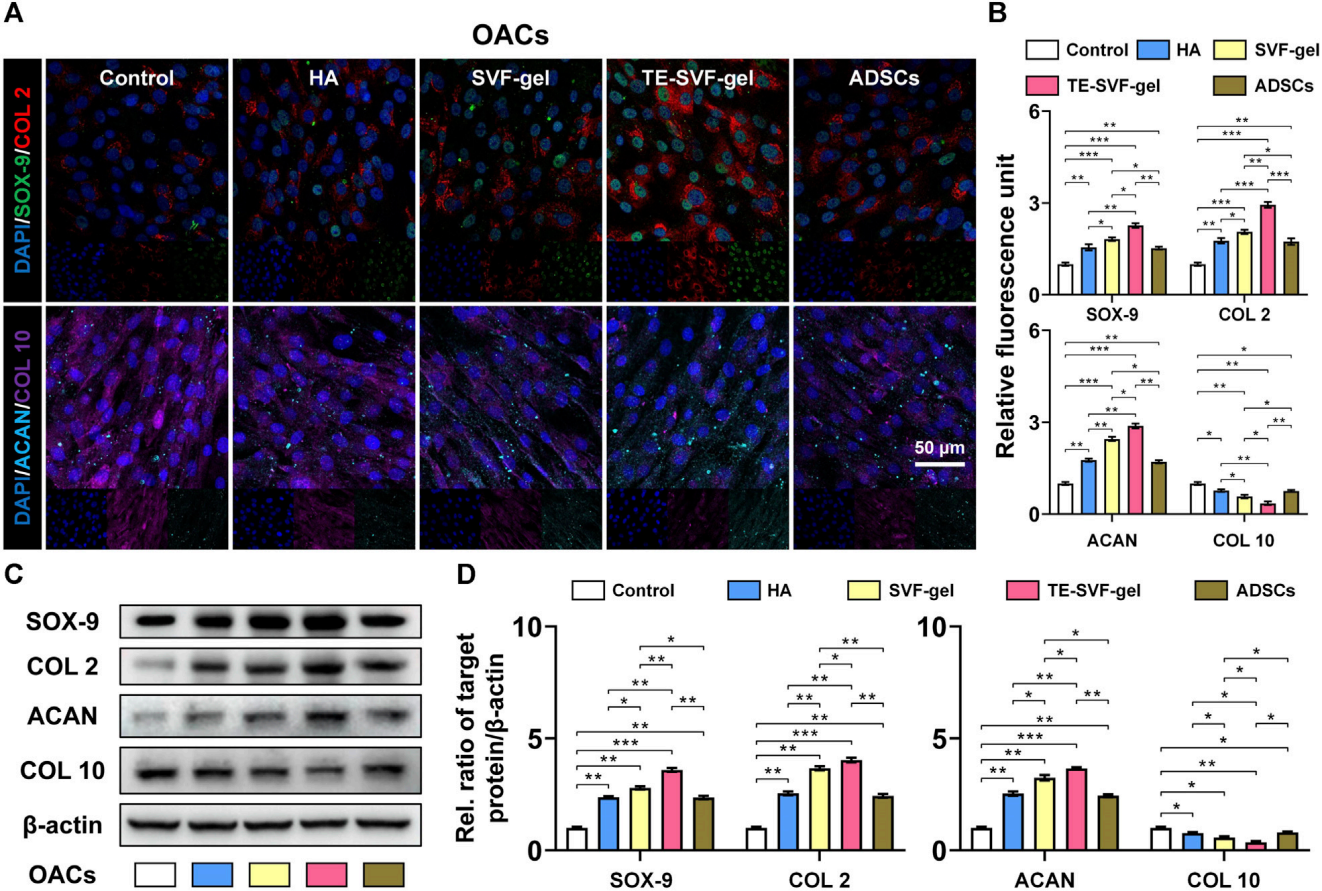
TE-SVF-gel effectively enhanced chondrogenic matrix expression and reduced hypertrophic differentiation in OACs. **(A)** Representative fluorescence images depicting the expression of COL 2, SOX-9, ACAN, and COL 10 in OACs; Nuclei were stained with DAPI; **(B)** The relative fluorescence unit of COL 2, SOX-9, ACAN, and COL 10 was analyzed. **(C)** Representative western blots of COL 2, SOX-9, ACAN, and COL 10 (OACs). **(D)** Statistical quantitative analysis of western blots (SOX-9, COL 2, ACAN, and COL 10). β-actin was served as a loading control for western blots. *: *p* < 0.05; **: *p* < 0.01; ***: *p* < 0.001.

In the indirect co-culture system, as showed in Western blotting and IF staining (Figures 2A–D), the SVF-gel effectively promoted the expression of COL 2, SOX-9, and ACAN in rat OACs, compared to the ADSCs and HA group, and reduced the expression of COL 10 (*p* < 0.05). Moreover, the TE-SVF-gel group showed even more enhanced expression of COL 2, SOX-9, and ACAN (*p* < 0.05), and lesser expression of COL 10 compared to the SVF-gel group (*p* < 0.05).

### 3.3 TE-SVF-gel mitigated pathological changes in rat DMM-induced OA model

**FIGURE 3:**
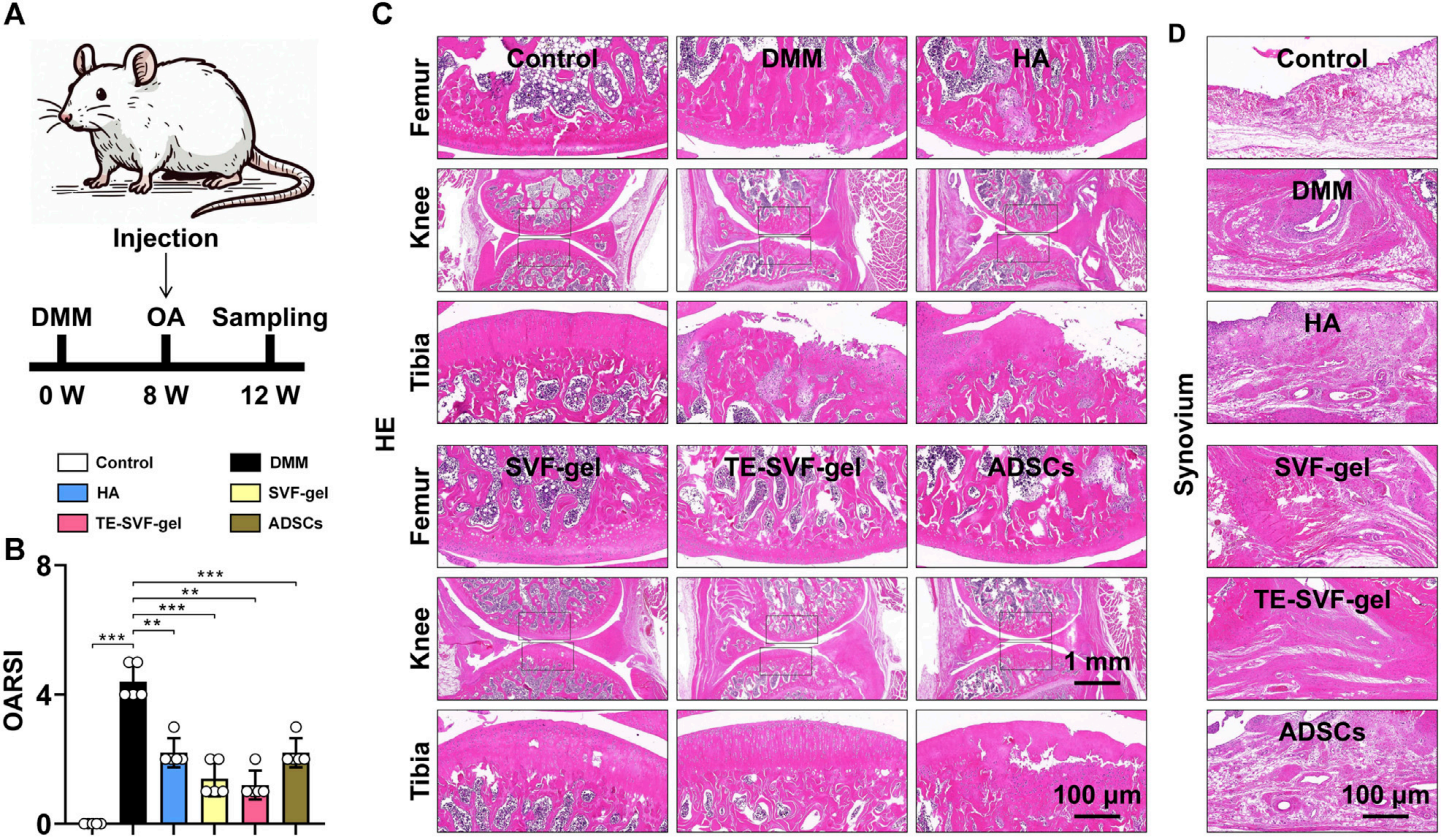
Intra-articular injection of TE-SVF-gel attenuates the progression of knee OA in rats. **(A)** Schematic showing the surgical and intra-articular injection protocol of OA rat with DMM surgery (black arrows indicate time points when intra-articular injection was performed). **(B)** OARSI score. **(C)** HE staining of rat knee joints (images of femur and tibia are from black boxed area). **(D)** HE staining of synovial tissue in the knee joint. **: *p* < 0.01; ***: *p* < 0.001.

The animal experiment workflow was shown in Figure 3A. Histological evaluation using HE staining revealed distinctive osteoarthritic features in the DMM group, such as disorganization of chondrocyte arrangement and degeneration of the cartilage matrix, compared to the normal group. This was further confirmed by a significantly higher OARSI score in the DMM group. Although the HA, SVF-gel, TE-SVF-gel, and ADSCs groups all showed reduction in OARSI scores compared to the DMM group, no significant differences were observed among these intervention groups (Figure 3B). In the HE staining, both the HA and ADSCs groups exhibited histological improvements compared to the DMM group, characterized by more organized chondrocytes, reduced signs of cartilage degeneration, and a smoother cartilage surface. The SVF-gel group demonstrated further enhancements in chondrocyte organization, decreased cartilage matrix degeneration, and an even smoother cartilage surface when compared with the HA and ADSCs groups. Notably, the TE-SVF-gel group displayed histological changes suggesting potential therapeutic benefits, including a chondrocyte arrangement approaching normal tissue patterns and minimal cartilage degeneration (Figure 3C). Examination of HE-stained synovial tissues revealed significant synovitis in the OA group compared to the control group, characterized by extensive inflammatory cell infiltration and increased thickness of the synovial lining. Following treatment, clear improvements in synovial inflammation were observed in the HA, SVF-gel, TE-SVF-gel, and ADSCs groups relative to the OA group; this was evidenced by decreases in inflammatory cell numbers and reduction in synovial lining thickness. Moreover, none of these four treatment groups showed any evident neovascular changes within their synovium as typically indicated by increased angiogenesis or vascular tissue proliferation highlighted through new blood vessel-like structure formation on HE staining. The level of improvement observed among these treatment groups was consistent without any significant differences noted (Figure 3D).

**FIGURE 4:**
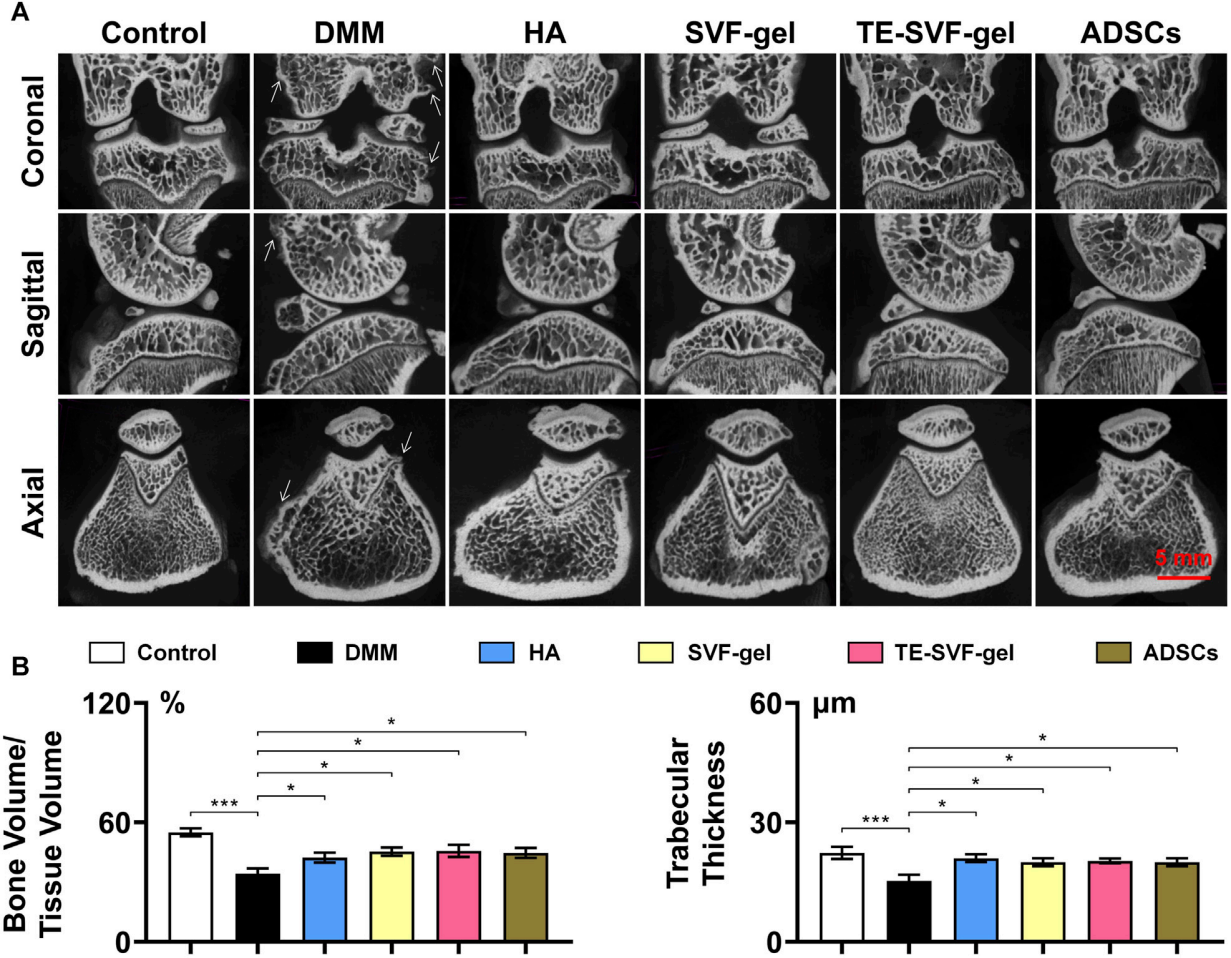
Intra-articular injection of TE-SVF-gel restored the bone structure in a OA rat model. **(A)** Micro-CT images of OA knee joints after single injections of HA, SVF-gel, TE-SVF-gel, or ADSCs. **(B)** Statistic analysis of Bone value/Tissue value and Trabecular thickness. *: *p* < 0.05; ***: *p* < 0.001.

Micro-CT scans (Figures 4A, B) of the knee joints in the DMM group revealed characteristic osteoarthritic changes, such as osteophyte formation and a significant decrease in BV/TV and trabecular thickness. The HA, SVF-gel, TE-SVF-gel, and ADSCs groups all demonstrated improved BV/TV, suggesting therapeutic effect on bone structure. However, there were no significant differences observed among these treatment groups. Similarly, all treatment groups showed reduced osteophyte formation compared to the OA group, but no significant differences were noted between the groups.

### 3.4 TE-SVF-gel increased level of proteoglycan and collagen matrix in OA rats

**FIGURE 5:**
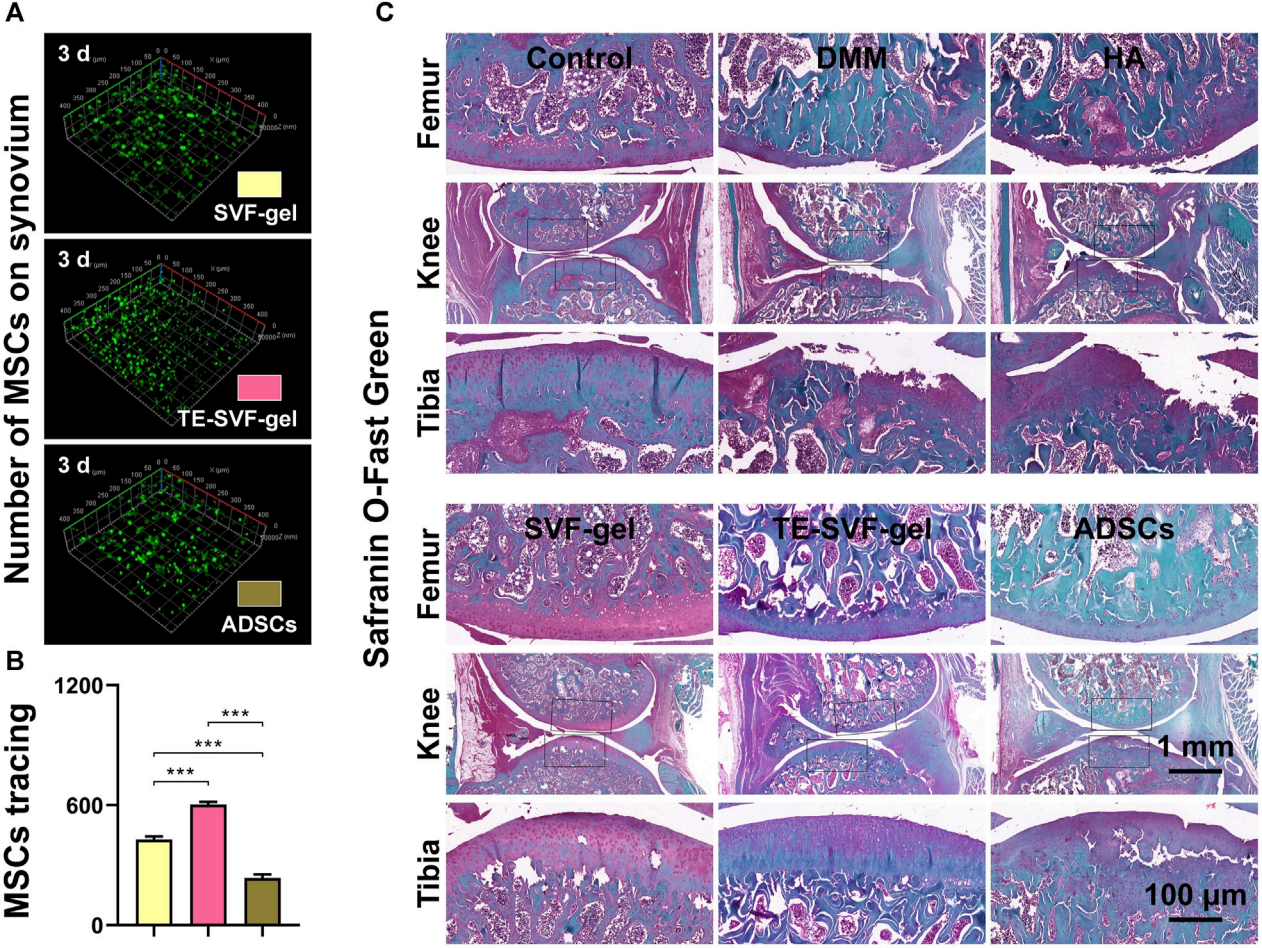
Intra-articular injection of TE-SVF-gel effectively protected the survival of ADSCs within the joint cavity and enhanced cartilage matrix expression in OA rats. **(A)** CD90 staining showing ADSCs attached to the synovium in the rat knee joint. **(B)** Quantitative analysis of CD90-positive ADSCs adhesion on the rat synovium. **(C)** Safranin O/Fast Green staining of rat knee joints. ***: *p* < 0.001.

MSCs tracking experiments were conducted to examine the quantity of MSCs on the synovial surface in the SVF-gel, TE-SVF-gel, and ADSCs groups. The results indicated that the TE-SVF-gel group showed the largest number of ADSCs, followed by the SVF-gel group, while the ADSCs group showed the least ADSCs (Figures 5A, B). In the DMM group, histological staining revealed reduced proteoglycan content and increased collagen matrix compared to the normal group. The HA and ADSCs groups showed restoration of proteoglycan content and decline in collagen matrix. The SVF-gel group exhibited even greater restoration. The most robust restoration was observed in the TE-SVF-gel group, demonstrating substantial recovery in proteoglycan content and normal collagen matrix, affirming its optimal therapeutic efficacy for OA (Figure 5C).

### 3.5 TE-SVF-gel reduced gastrocnemius muscle atrophy in lower limbs and enhanced expression of COL 2 in articular cartilage of OA rats

**FIGURE 6:**
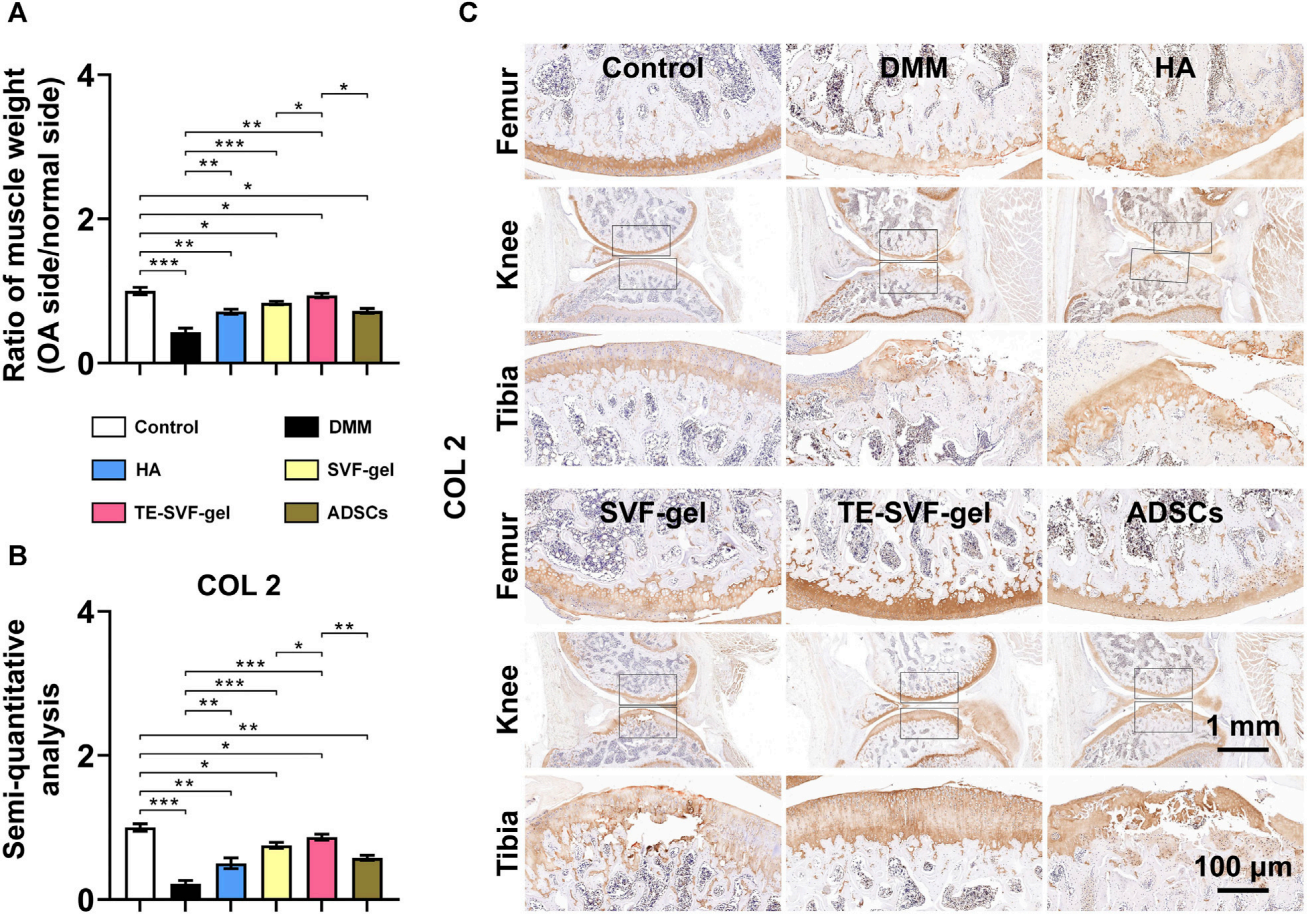
Intra-articular injection of TE-SVF-gel reduced gastrocnemius muscle atrophy in lower limbs and increased expression of COL 2 in articular cartilage of OA rats. **(A)** Ratio of gastrocnemius muscle weight (OA side/normal side). **(B)** IHC staining of COL 2 in rat knee joints. **(C)** Semi-quantitative analysis of COL 2 IHC staining. *: *p* < 0.05; **: *p* < 0.01; ***: *p* < 0.001.

In response to the significant muscle atrophy that occurred in OA-affected limbs due to pain, muscle weight was also measured in our study. As expected, the OA group exhibited substantial muscle atrophy on the OA-affected limb (Figure 6A). In contrast, we observed a varying degree of muscle weight recovery across all treatment groups. In terms of COL 2 expression, the OA group showed a marked decrease compared to the normal group, indicative of cartilage degradation. Both the HA and ADSCs groups demonstrated an increase in COL 2 staining, suggesting a recovery in cartilage matrix synthesis. The SVF-gel group showed a further increase in COL 2 staining, indicating more robust cartilage regeneration. The most pronounced COL 2 expression was observed in the TE-SVF-gel group, almost comparable to the normal group, suggesting near-normal cartilage synthesis. These findings, together with the recovery of muscle weight, further corroborate the superior therapeutic efficacy of TE-SVF-gel for OA (Figures 6B, C).

## 4 Discussion

The origin and progression of OA involve intricate mechanisms that are still not fully understood. OA primarily affects the articular cartilage, contributing to its continual degradation, the subsequent formation of osteophytes, and increased subchondral bone sclerosis. The results of this study highlight the potential of TE-SVF-gel in mitigating the pathological alterations observed in an OA rat model. This novel approach, which utilizes the regenerative properties of ADSCs within the gel, indicates promising possibilities for OA treatment.

MSCs therapies for OA often involve physiological saline or HA as solvent (Lopa et al., 2019). In these solutions, MSCs exist in a suspended, free-floating state without ECM support. Upon injection into the joint, cell survival depends on their adhesion to the host tissue. This process can be challenging as cells may experience anoikis, a form of programmed cell death that occurs when anchorage-dependent cells detach from the surrounding ECM (Eseonu and De Bari, 2015; Markov et al., 2021). The SVF-gel, however, provides direct ECM support for stem cells, reducing the likelihood of cell death due to detachment or “cellular homelessness.” The gel matrix in SVF-gel creates a conducive environment that promotes cell adhesion, proliferation, and differentiation, enhancing their survival and function (Jiang et al., 2020). This approach offers a promising alternative to MSCs based interventions for OA. Our experimental results demonstrated the superior efficacy of SVF-gel over traditional MSCs therapies, as evidenced by that SVF-gel enhanced both the survival and retention of the injected cells within the joint. Furthermore, it contributed to significant improvements in joint morphology and function in the OA rat model. These improvements were significantly greater than those observed in the group treated with MSCs alone. The results supported the hypothesis that the SVF-gel, by providing an immediate ECM support to the injected cells, can enhance the therapeutic effects of stem-cell based interventions for OA. Therefore, SVF-gel presents a compelling alternative to MSCs therapies in treating OA, potentially offering more effective relief from the debilitating symptoms of this chronic condition.

Biomaterials have shown promising advancements in the field of OA, with a variety of materials developed for treating animal models of OA to notable effect (Li et al., 2023). For example, a lyotropic liquid crystal (LLC) precursor-based gel, co-loaded with HA and celecoxib, which provides sustained OA treatment through anti-inflammatory action and cartilage protection while maintaining biocompatibility and stress absorption (Mei et al., 2021). Besides, Priya Katyal et al. developed a protein-based E5C gel that forms a network at physiological temperatures for sustained delivery of the chondroprotective agent Atsttrin, demonstrating both preventive and therapeutic effects against post-traumatic osteoarthritis in a rabbit model (Katyal et al., 2022). In addition, in a study comparing ibuprofen gel and cream phonophoresis for knee OA, gel phonophoresis demonstrated significantly greater improvements in pain and functionality as measured by VAS and WOMAC scores (Coskun Benlidayi et al., 2018). Moreover, a silk fibroin hydrogel crosslinked with diglycidyl ether, demonstrating high elasticity and anti-fatigue properties suitable for OA treatment, with *in vivo* tests confirming its biocompatibility and potential for pain relief and sustained drug delivery. Compared to these biomaterials, SVF-gel, with its simple preparation process, autologous source minimizing immunogenicity, and exceptional biosafety profile, stands out as a cost-effective and accessible option. Due to its cost-effectiveness and ease of production, SVF-gel is highly suitable for widespread implementation in primary care settings. With significant potential for clinical translation and straightforward quality control, SVF-gel presents a promising novel alternative for the treatment of OA.

In the process of preparing SVF-gel, the adipose tissue undergoes mechanical disruption by rotating blades, a process designed to break down fat cells and extract the stromal vascular fraction. Lasting for 10–20 s, this process generates heat and shearing forces that exert damaging effect on embedded cells. Our experimental results corroborate this, as we observed an approximately 7.91% cell mortality rate within the SVF-gel, indicating cellular injury during the preparation. To mitigate this damage and protect the cells within the SVF-gel, we introduced TE, a soluble ECM protein. TE can offer a form of ECM, creating a supportive environment for the cells and potentially alleviating the effects of anoikis, a form of cell death that occurs when cells lose contact with the ECM (Wise et al., 2014; Yeo and Weiss, 2019). By providing an immediate ECM-like environment, TE can potentially shield the cells from the stress and damage induced by the SVF-gel preparation process. Furthermore, it may enhance the vitality and functionality of the cells within the SVF-gel. As a result, the strategy of combining SVF-gel with TE could offer a more effective alternative for cell therapies in OA, augmenting the therapeutic potential of the treatment.

In our animal experiments, we demonstrated that SVF-gel exerted excellent injectability, matching the smoothness of HA injections. This ease of administration underscores the practicality of SVF-gel as a treatment medium. Importantly, despite having fewer stem cells than ADSCs, SVF-gel outperformed both HA and ADSCs in therapeutic efficacy. Our MSCs tracking experiments further corroborate this finding. We observed that the TE-SVF-gel group exhibited the highest quantity of MSCs on the synovial surface, followed by the SVF-gel group, while the ADSCs group had the least. This suggested that TE-SVF-gel and SVF-gel provided a more conducive environment for MSCs retention and survival. The SVF-gel treatment led to an enhanced synthesis of cartilage matrix, mitigated the formation of osteophytes, and promoted the expression of COL 2. Many studies have shown that the majority of ADSCs tend to disappear within about 7 days of being injected into the joint cavity. Therefore, the superior performance of SVF-gel could be attributed to its ECM, which provided a supportive environment that mitigates anoikis, enabling MSCs to survive and function more effectively. Our results were further improved with the addition of TE to the SVF-gel. As we have seen in previous research, TE contributed to the early adhesion, migration, differentiation, and paracrine activity of MSCs. Therefore, the combined use of SVF-gel and TE not only provides a viable and effective treatment strategy for OA but also maximizes the therapeutic potential of MSCs.

The potential of SVF-gel for future clinical applications appears promising. Of particular note, preparing SVF-gel involves sourcing adipose tissue from subcutaneous fat of the patient, a process that is minimally invasive and carries low risk. An added advantage is the ability of SVF-gel to be stored at low temperatures for an extended period, allowing for a single extraction that requires only a small incision (Tao et al., 2023). Another significant aspect to consider is the adaptability of SVF-gel for use in clinical surgeries. For instance, during hip arthroscopy (Jamil et al., 2018), the path to the hip joint is established subcutaneously, where adipose tissue can be harvested and processed into SVF-gel. This SVF-gel can then be injected to repair acetabular labrum injuries. The same principle applies to knee arthroscopy (Nyland et al., 2015), where the infrapatellar fat pad serves as a source of adipose tissue (Dragoo et al., 2012). This fat pad, a known source of inflammatory factors within the joint cavity (Zeng et al., 2020), can be transformed from “waste” into a therapeutic tool through its conversion into SVF-gel and subsequent injection into the joint cavity. Moreover, the preparation of SVF-gel is relatively quick, typically completed within 20–30 min (Cai et al., 2020), making it feasible to prepare during arthroscopic surgeries. These qualities enhance the adaptability and potential of SVF-gel in various surgical scenarios, further expanding its therapeutic utility in OA treatment.

While the findings of this study lend promising insights into the potential use of TE-SVF-gel in mitigating osteoarthritic changes, a few limitations should be acknowledged. Firstly, the use of a rat model, though commonly employed, may not entirely represent the intricacies of human OA. Differences in joint anatomy, loading conditions, and disease progression between humans and rodents can potentially limit the direct applicability of our findings to human patients. Secondly, the duration of our study may not fully capture the long-term efficacy and safety of TE-SVF-gel treatment. Thirdly, accurately characterizing the composition of TE-SVF-gel is a notable challenge. The difficulty in quantifying the cellular, extracellular matrix, and soluble factor components of the concentrate may introduce variability during clinical trials. This could impact the assessment of clinical outcomes and the ability to draw definitive conclusions. Standardizing these measurements will be crucial for the clinical application of TE-SVF-gel in osteoarthritis therapy. Fourthly, considering the potential pro-angiogenic effects of TE (Mithieux et al., 2018; Al Halawani et al., 2021)^,^ our study indicates the need for further investigation into the long-term angiogenic outcomes and the effects of higher doses of TE in the treatment of OA. Future animal experiments are required to substantiate these aspects. As OA is a chronic disease that progresses over a prolonged period, future studies with an extended duration are essential to confirm if the beneficial effects of TE-SVF-gel persist over time. These limitations provide critical directions for future research in the therapeutic application of TE-SVF-gel for OA treatment.

## 5 Conclusion

In conclusion, our study provides substantial evidence that supports the therapeutic potential of TE-SVF-gel for the treatment of OA. This presents a promising and possibly more effective, less invasive option for OA treatment compared to current methods. However, to fully leverage this promising therapeutic strategy, further studies are required to better understand the underlying mechanisms and long-term outcomes of using TE-SVF-gel in OA treatment.

## Data Availability

The raw data supporting the conclusion of this article will be made available by the authors, without undue reservation.
